# Peptide Blockers of PD-1-PD-L1 Interaction Reinvigorate PD-1-Suppressed T Cells and Curb Tumor Growth in Mice

**DOI:** 10.3390/cells13141193

**Published:** 2024-07-15

**Authors:** Shanshan (Jenny) Zhong, Xiaoling Liu, Tomonori Kaneko, Yan Feng, Owen Hovey, Kyle Yang, Sally Ezra, Soon-Duck Ha, Sung Kim, John K. McCormick, Huadong Liu, Shawn Shun-Cheng Li

**Affiliations:** 1 Department of Biochemistry, Schulich School of Medicine and Dentistry, Western University, London, ON N6A 5C1, Canada; szhong43@uwo.ca (S.Z.); liuxiaoling0508@gmail.com (X.L.); tkaneko@uwo.ca (T.K.); yfeng562@uwo.ca (Y.F.); ohovey@uwo.ca (O.H.); kyang59@uwo.ca (K.Y.); sally.ezra@albertaprecisionlabs.ca (S.E.); 2 Department of Microbiology and Immunology, Schulich School of Medicine and Dentistry, Western University, London, ON N6A 5C1, Canada; sha3@uwo.ca (S.-D.H.); skim283@uwo.ca (S.K.); john.mccormick@uwo.ca (J.K.M.); 3 School of Health and Life Sciences, Health and Rehabilitation University, 369 Dengyun Rd, Qingdao 266071, China; huadongliu@outlook.com

**Keywords:** programmed cell death-1 (PD-1), PD-1 ligand-1 (PD-L1), immune checkpoint blockade, PD-1/PD-L1 blocking peptide, peptide-based immunomodulator

## Abstract

The programmed cell death protein 1 (PD-1) plays a critical role in cancer immune evasion. Blocking the PD-1-PD-L1 interaction by monoclonal antibodies has shown remarkable clinical efficacy in treating certain types of cancer. However, antibodies are costly to produce, and antibody-based therapies can cause immune-related adverse events. To address the limitations associated with current PD-1/PD-L1 blockade immunotherapy, we aimed to develop peptide-based inhibitors of the PD-1/PD-L1 interaction as an alternative means to PD-1/PD-L1 blockade antibodies for anti-cancer immunotherapy. Through the functional screening of peptide arrays encompassing the ectodomains of PD-1 and PD-L1, followed by the optimization of the hit peptides for solubility and stability, we have identified a 16-mer peptide, named mL7N, with a remarkable efficacy in blocking the PD-1/PD-L1 interaction both in vitro and in vivo. The mL7N peptide effectively rejuvenated PD-1-suppressed T cells in multiple cellular systems designed to recapitulate the PD-1/PD-L1 interaction in the context of T-cell receptor signaling. Furthermore, PA-mL7N, a chimera of the mL7N peptide coupled to albumin-binding palmitic acid (PA), significantly promoted breast cancer cell killing by peripheral blood mononuclear cells ex vivo and significantly curbed tumor growth in a syngeneic mouse model of breast cancer. Our work raises the prospect that mL7N may serve as a prototype for the development of a new line of peptide-based immunomodulators targeting the PD-1/PD-L1 immune checkpoint with potential applications in cancer treatment.

## 1. Introduction

T-cell function is regulated by a balance between activating and inhibitory signals, the latter of which include those mediated by immune checkpoints [1]. Of note, the binding of programmed cell death-1 (PD-1) by its ligand (PD-L1) triggers a co-inhibitory signaling pathway that suppresses T-cell activation, contributing to cancer immune evasion [2]. PD-1 (also known as CD279) is a type I transmembrane receptor expressed on the surface of several cell types, including T cells, B cells, monocytes, natural killer cells, and dendritic cells [3]. PD-1 binds to and is activated by two naturally occurring ligands, PD-L1 (B7-H1, CD274) and PD-L2 (B7-DC, CD273) [4]. Like PD-1, PD-L1 and PD-L2 are Ig-like transmembrane proteins that are expressed on the cell surface [5]. PD-L1 is expressed in both hematopoietic and non-hematopoietic cells, and its expression may be regulated by extrinsic stimuli, including signals emanating from Toll-Like Receptors (TLRs) or Interferon-gamma (IFN-γ) [6]. PD-L2 is normally expressed on the surface of macrophages, dendritic cells, mast cells, and certain B cells [5]. The crystal structure of a mouse PD-1/human PD-L1 (mPD-1/hPD-L1) complex [7] has revealed the molecular basis of the PD-1-PD-L1 interaction, suggesting that the interaction may be disrupted by either PD-1- or PD-L1-specific antibodies or peptides that target the binding interface of the two proteins.

Immunotherapies based on monoclonal antibodies that block the PD-1/PD-L1 interaction have transformed the treatment of certain cancers such as melanoma and non-small-cell lung cancer but yielded less favorable outcomes for other cancers, including cancers of the breast, colon, and pancreas [8]. The disparity in the efficacy of PD-1 or PD-L1 blockade antibodies in different types of cancer underscores the need to develop novel strategies of immunotherapy that may overcome the limitations of existing immunotherapeutic agents. In contrast to antibodies, which are large in size and difficult to produce, small molecules and low-molecular-weight peptides capable of blocking the PD-1/PD-L1 interaction provide an alternative that may be exploited as a unique line of immunotherapeutic agents. The latter may afford less immune-related toxicity, reduced immunogenicity, better tumor penetration, and lower manufacturing cost [9]. Despite these potential advantages, progress has been slow in developing peptide antagonists of PD-1 or PD-L1. Nevertheless, several low-molecular-weight peptides have been identified with promising in vitro and in vivo characteristics. By screening a phage-displayed peptide library for peptide inhibitors targeting PD-L1, Chang et al. identified several hydrolysis-resistant D-peptide antagonists and showed that ^D^PPA-1 effectively disrupted the PD-1/PD-L1 interaction in vivo [10]. Using a similar strategy, Liu et al. identified several peptides that could block the binding of PD-L1 to PD-1 in vitro and inhibit tumor growth in mice [9]. AUNP-12, a peptide-based therapeutic agent targeting PD-1 developed by Aurigene, has shown activity in promoting splenocyte proliferation [11]. By screening a bacterium-displayed peptide library, Li et al. identified TPP-1, a peptide that binds PD-L1 with a high affinity (Kd = 95 nM) [9]. T-cell activation and in vivo studies showed that TPP-1 could rejuvenate PD-1-exhausted T cells and serve as an alternative to anti-PD-L1 antibodies in curbing tumor growth in mice [12]. PL120131, a peptide designed to mimic the PD-1-binding pocket in PD-L1, has been shown to interfere with the PD-1-PD-L1 interaction and promote the anti-tumor activity of cytotoxic T cells [13]. Collectively, these studies have provided evidence in support of the notion that peptide blockers of the PD-1-PD-L1 interaction may be developed as alternatives to blockade antibodies for potential immunotherapy. 

Here, we report findings from functional screening of overlapping peptides representing the ectodomains of human PD-1 and PD-L1. Rather than screening for binders of PD-1 or PD-L1, we scrutinized the peptides for the ability to activate suppressed T cells in a coculture of PD-1-expressing Jurkat T cells (JT-PD-1) with Chinese hamster ovary (CHO) cells overexpressing PD-L1 and a T-cell receptor (TCR) activator (CHO-PDL1). Hit peptides identified from the functional screen underwent additional rounds of optimization to generate peptides with a desired physiochemical profile and biological activity. Our study yielded a peptide, named L7N, derived from the PD-L1 ectodomain, which exhibited potent ability to block the PD-1-PD-L1 interaction in vitro and in vivo, reinvigorate PD-1-suppressed T cells, and inhibit tumor growth in mice by promoting the tumor recruitment of CD8+ T cells. L7N, a 16-mer peptide, and its derivative may be explored as novel immunotherapeutic agents for cancer treatment by reinvigorating PD-1-exhausted T cells.

## 2. Materials and Methods

### 2.1. Peptide Synthesis

Peptides were synthesized on a Multipep synthesizer from Intavis AG Bioanalytical Instruments in a 96-well format at a 1 μm/well scale on TentaGel amide resin (Intavis Catalog #32.900, Charlotte, NC, USA) by 9-fluorenylmethyloxycarbonyl (Fmoc) chemistry. Upon completion of the synthesis, peptides were cleaved off the resin by incubating the peptide resin in a solution comprising 95% trifluoroacetic acid (TFA), 3% triisopropylsilane (TIPS), and 2% H_2_O. The cleaved peptides were purified by 3× cold-ether precipitation followed by desalting on Sephadex^®^ G10 (Sigma-Aldrich, St. Louis, MI, USA) columns or purification with HPLC (C18 columns; Waters) for peptides used in mouse studies. Peptide masses were validated by MALDI-TOF mass spectrometry [14]. 

Palmitic-acid-tagged peptides were synthesized following previously reported protocol [15]. Specifically, the peptide synthesis was extended at the N-terminus to include the tag sequence -ahx-EYEKEYE-ahx, ahx, denoting 6-aminohexanoic acid, and biotin for biotin pulldown experiments. Alternatively, the peptide synthesis was further extended at the N-terminus to include the tag sequence EYEKEYE-(PEG)_2_ (PEG)_2_ (PEG)_2_ for the efficacy test in vitro and in vivo. The Lys residue within the tag was introduced as Mtt-Lys during synthesis, and the Mtt protection group was subsequently removed by washing the peptide resin in 2% hydrazine hydrate in DMF three times, each for a duration of 3 min. Subsequently, the peptide was plamitoylated at the Lys sidechain by incubating the resin with free palmitic acid (at 2 equivalents), DCC (at 2 equivalents), and HOBt (at 4 equivalents) in 3 mL of DMF, followed by agitation at 400 r.p.m. for 1 h. Cleavage and purification of the PA-tagged peptides followed the same procedure as for non-tagged peptides.

### 2.2. Cell Culture

The Jurkat T cells overexpressing PD-1 and an NFAT reporter (JT-PD1, catalog#: 60535) and CHO cell line overexpressing PD-L1 and TCR activator (CHO-PDL1, catalog#: 60535) were obtained from BPS Bioscience. The hPD-L1-overexpressing Raji B cell line (Raji-PDL1) was obtained from InvivoGen. JT-PD1 cells were cultured in RPMI-1640 medium supplemented with 10% FBS, 1 mg/mL geneticin, and 0.2 mg/mL hygromycin B. CHO-PDL1 cells were grown in Ham’s F-12 medium containing 10% FBS, 1 mg/mL of geneticin, and 0.4 mg/mL of hygromycin B. Raji-PDL1 cells were cultured in IMDM medium with 2 mM L-glutamine, 25 mM HEPES, 10% heat-inactivated fetal bovine serum (FBS), and 10 μg/mL blasticidin. 

### 2.3. Coculture of JT-PD1 Cells with CHO-PDL1 or Raji-PDL1 Cells

CHO-PDL1 cells were seeded at a density of 35,000 cells per 100 μL of culture medium in a 96-well microplate and incubated overnight at 37 °C. On the next day, JT-PD1 cells, resuspended in RPMI-1640 medium without selective antibiotics, were added to the wells at 20,000 cells/well. JT-PD1 or CHO-PDL1 cells were pretreated with peptide inhibitors for 30 min before mixing the two cell lines. The plates were incubated in a CO_2_ incubator at 37 °C for 5–6 h for the NFAT luciferase assay. 

For the luciferase assay, 100 µL of One-Step Luciferase reagent (BPS Bioscience, San Diego, CA, USA) was added to each well, and the plate was gently agitated at room temperature for 15 min. A BioTek Cytation 1 Cell Imaging Multimode Reader (Agilent Technologies, Inc., Santa Clara, CA, USA) was used to quantify luminescence. The average background luminescence (from the cell-free control wells) was subtracted from the luminescence reading of the sample wells. The fold induction of NFAT luciferase reporter expression was calculated by dividing the background-subtracted luminescence of the treated wells by the average background-subtracted luminescence of the control wells.

For Raji-PDL1 and JT-PD1 coculture, Raji cells were incubated with 10 ng/ml staphylococcal enterotoxin E (SEE) at 37 °C for 30 min. After priming Raji-PDL1 cells with SEE, the cells were washed three times with warm PBS. In a clear-bottom 96-well microplate, 2 × 10^5^ SEE-treated Raji-PDL1 cells (100 μL) were seeded with JT-PD1 cells in a 1:1 ratio. Following centrifuging the plate to spin down the mix of two cell lines, the plate was incubated in a CO_2_ incubator at 37 °C to initiate the interaction of the two cell lines. After coculturing for 24 h, 100 μL of supernatant was taken for IL-2 measurement using the ELISA MAX Deluxe SET Human IL-2 (BioLegend, San Diego, CA, USA). 

### 2.4. Immunoprecipitation (IP) and Western Blotting (WB)

Cells or tumor tissues were lysed in a buffer composed of 20 mM Tris-HCL (pH 7.4), 150 mM NaCl, 1 mM EDTA, 1% Triton-X100, 1% sodium deoxycholate, 0.1% SDS, and the cOmplete™ Protease Inhibitor Cocktail (SKU: 11697498001). The cell lysate was then sonicated on ice for 30 s, while the tumor lysate was fully homogenized on ice. Subsequently, the lysate was centrifugated at 14,000× *g* for 20 min at 4 °C, and the resulting supernatant was collected. The protein concentration in the supernatant was determined using the BCA protein assay (Thermo Fisher Scientific, Waltham, MA, USA; Catalog #: 23225). A 20 μg portion of the whole cell lysate was loaded into each well and subjected to electrophoresis on an 8–12% polyacrylamide gel (PAGE). Following electrophoresis, the proteins were transferred to a PVDF membrane and subsequently subjected to immunoblotting using the designated primary antibody at a dilution of 1:1000–1:2000, incubated overnight at 4 °C. The blot was then incubated with diluted (1:5000–1:10,000 dilution) fluorescence-labeled secondary antibodies. The quantification of protein bands on Western blots was performed using the ImageLab4.1 software. Relative protein levels were estimated by normalizing band intensities to either whole protein or GAPDH levels.

For immunoprecipitation (IP) [14], 300 µg of lysate was mixed with 5 µg of human anti-PD-1 (#AF1086, R&D) or mouse anti-PD-L1 (#PA5-20343, Invitrogen-Thermo Fisher Scientific) at 4 °C for 2 h followed by 30 min incubation with 20 µL of Pierce™ Protein A/G Magnetic Beads (ThermoFisher Scientific; catalog #88802) at room temperature followed by Western blot to detect the interacted proteins. In the biotin pulldown procedure, 1 mM biotinylated peptides were incubated with 0.03 mM of albumin (BSA, HSA, the equivalent amount FBS) for 2 h at 4 °C followed by incubating with 50 µL Streptavidin resin (Genescript, Piscataway, NJ, USA) for 1 hr at room temperature, followed by SDS-PAGE to detect the bound albumin.

### 2.5. Mouse Experiments

Mouse experiments were conducted in accordance with an approved animal care and use protocol (AUP2022-138) of Western University. To establish tumors, 4T1 cells (5 × 10^4^ cells/mouse) were resuspended in serum-free medium mixed with an equal volume of Matrixgel Basement Membrane Matrix (BD Biosciences, San Jose, CA, USA), followed by injection into the mammary fat pad of female BALB/c mice aged 6–8 weeks from Charles River. On day 7 following the inoculation of 4T1 cells, mice received daily injections of either control peptides or peptide inhibitors at varying doses via intraperitoneal (i.p.) injection for 2–3 weeks. For comparison, groups of mice were administered (via i.p.) either an IgG isotype control (Clone 2A3, Bio X Cell, Lebanon, NH, USA) or anti-PD-1 antibody (Clone RMP1-14, Bio X Cell) at 1 mg/kg on days 7, 10, 13, 16, and 19 post 4T1 cell inoculation. Tumor size was measured using a caliper every three days, and tumor volume was calculated and represented graphically. At the conclusion of the treatment period, tumor tissues were collected for further analysis.

#### Flow Cytometry

At the endpoint of treatment (typically 21 days post tumor cell inoculation), tumors were harvested. The excised tumors underwent a digestion process utilizing 10% collagenase/hyaluronidase (Stem cell Technologies, Vancouver, BC, Canada) and 0.15 mg/mL DNase (Sigma-Aldrich, St. Louis, MI, USA) in RPMI 1640 medium at 37 °C for 30 min. The digested tumor tissues were subsequently passed through a 70 μm nylon mesh strainer to obtain a single-cell suspension. The cells were subsequently stained with specific markers for tumor-infiltrating lymphocytes (TILs) by gating the total immune cell with Super Bright^TM^ 702 CD45 antibody (# 67-0451-82, Invitrogen), lymphocytes with Alexa Fluor^®^ 488 anti-mouse CD3 antibody (#100212, BioLegend), and the CD8+ T cells with Alexa Fluor^®^ 594 anti-mouse CD8a antibody (#100758, BioLegend). The discrimination between live and dead cells was accomplished using the Zombie NIR viability dye following the Biolegend staining protocol. The stained samples were then subjected to analysis using an LSR II SORP Cytometer from BD Biosciences, with data acquisition performed through the BD FACSDiVa software v.6.1.3. Data analyses were carried out using Flowjo (v.10).

## 3. Results

### 3.1. Identification of PD-1/PD-L1 Blockade Peptides by Functional Screening of Peptide Libraries

**Figure 1:**
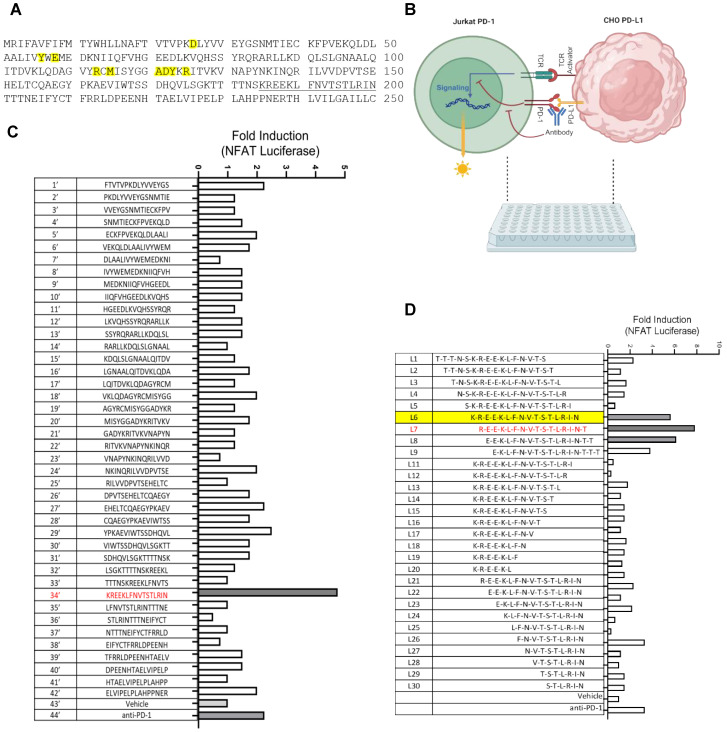
Peptide array combined with functional screen led to the identification of PD-1/PD-L1-blocking peptides. (**A**) The amino acid sequence of human PD-L1 ectodomain. Residues involved in PD-1 binding are highlighted in yellow. The underlined sequence corresponds to peptide No. 34′ in the walking array below. (**B**) Peptides were assayed for the ability to reactivate PD-1-suppressed T cells based on NFAT luciferase signals in coculture of PD-1-expressing Jurkat T cells (JT-PD1, which also expresses an NFAT luciferase reporter) and PD-L1-expressing CHO cells (CHO-PDL1, which also expresses a TCR activator). (**C**) Bar graph demonstrating relative efficacy of PD-L1-derived peptides in increasing NFAT transcription (shown in fold change over the DMSO vehicle control) in the JT-PD1-CHO-PDL1 coculture. (**D**) Peptide L7, derived from PD-L1 truncation array, effectively activated T cells in the coculture. An anti-PD-1 antibody (αPD-1, 2.5 μg/mL) was included in the control. L6, highlighted in yellow, was equivalent to 34′ in panel (**C**). Peptide concentration used in panels (**C**,**D**): 200 μM.

Because the PD-1-PD-L1 interaction is mediated by the extracellular domains of the two proteins, we predicted that certain peptides derived from the corresponding regions in PD-1 or PD-L1 might be able to block their interaction. To explore this possibility, peptide walking arrays representing the ectodomains of human PD-1 and PD-L1, each 16–18 amino acids in length and with a 3- or 5-residue overlap between consecutive peptides, were synthesized via parallel solid-phase peptide synthesis on 96-well plates (Figure 1 and Figure S1). The resulting peptides were subsequently evaluated for the ability to activate PD-1/PD-L1-suppressed T cells in an artificial T cell–antigen presenting cell (APC) coculture. Specifically, Jurkat T cells overexpressing PD-1 and firefly luciferase under the control of the NFAT promoter (JT-PD1) were cocultured with CHO cells overexpressing PD-L1 and a TCR activator. The engagement of PD-1 by PD-L1 leads to the suppression of T cells, which can be released by a PD-1 or PD-L1 blocking antibody. By replacing the PD-1/PD-L1 antibody with a peptide in the coculture, one could gauge the ability of the peptide to reactivate suppressed T cells by measuring the luciferase reporter signal (Figure 1B).

The first array of peptides representing the ectodomain of human PD-1 was synthesized as overlapping 18 mers (Figure S1A,B). Individual peptides from the array were evaluated with the JT-PD-1/CHO-PDL1 coculture, demonstrating that peptide No. 12 effectively promoted NFAT transcription in T cells (Figure S1C). The sequence of this peptide encompassed several residues involved in PD-1 binding to PD-L1 (Figure S1A). To define the minimum sequence required for efficacy, we synthesized arrays of peptides encompassing peptide No. 12 and the flanking regions as 18 mers with a three-residue overlap. We also synthesized arrays of peptides in which peptide No. 12 was truncated from the N- or C-terminus, one residue at a time (Figure S2A). Screening of this secondary peptide array led to the identification of peptide #26, which exhibited a greater efficacy than anti-PD-1 in reactivating PD-1-suppressed T cells in our coculture assay (Figure S2B). Intriguingly, the N-terminal half of peptide #26 is identical to the effective peptide segment in AUNP-12, a peptide-based immune checkpoint modulator developed by Aurigene [16], underscoring the strength of our approach in identifying promising peptide blockers of PD-1/PD-L1.

A similar strategy was applied to the human PD-L1 to identify peptides that could reinvigorate PD-1-suppressed T cells. To this end, 42 peptides representing the PD-L1 ectodomain were synthesized and subjected to functional screens utilizing the JT-PD1/CHO-PDL1 coculture, which led to the identification of peptide No. 34′ as a lead (Figure 1C). A secondary peptide walking array focused on the region encompassing the No. 34′ peptide and a truncation peptide series were synthesized (Figure 1D). Subsequent functional screens yielded peptide L7 (REEKLFNVTSTLRINT) as a potent activator of PD-1-suppressed T cells (Figure 1D). Indeed, L7 was more effective than a PD-1 antibody in our functional assay. Intriguingly, L7 (or No. 34′), derived from the IgC domain of PD-L1, is located distal from the PD-1-binding interface based on the PD-1/PD-L1 complex structure (Figure 1A).

### 3.2. Identification of Peptide L7N with Enhanced Solubility and Efficacy

**Figure 2:**
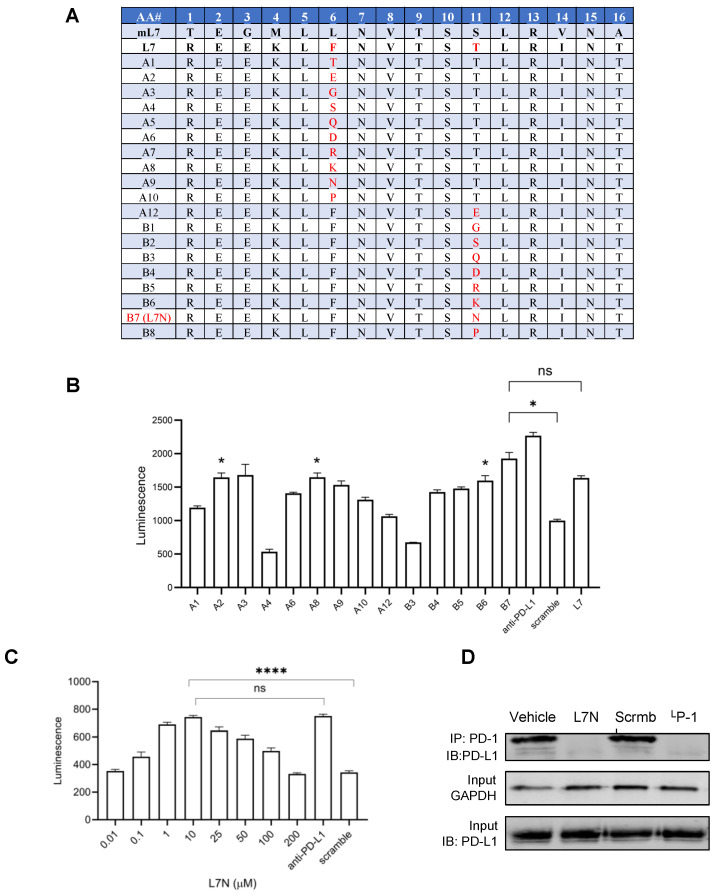
Optimization of efficacy and solubility for L7. (**A**) L7 analogues (from human PD-L1) generated by substituting the non-conserved hydrophobic residues (i.e., AA #6 and #11) with hydrophilic residues. mL7, an analogue of L7 corresponding to mouse PD-L1. (**B**) Efficacy of L7 analogues from (**A**) in activating PD-1-suppressed T cells. Shown are the NFAT luciferase signals in the presence of 50 μM peptide. L7 and anti-PD-L1 antibody (5 μg/mL) were included as a positive control, while a scrambled L7 peptide was included as a negative control. *, *p* < 0.01 compared to the scrambled peptide; ns, not significant; one-way ANOVA test. (**C**) Dose response of peptide L7N determined using the same assay as in (**B**). The optimal concentration for L7N was 10 μM. ****, *p* < 0.00001; one-way ANOVA test. (**D**) L7N, but not its scrambled version, effectively blocked the PD-1-PD-L1 interaction in the JT-PD1/CHO-PDL1 coculture. The positive control peptide, ^L^P-1, was based on Chang et al. (reference [10]). Concentration of peptides used: 10 μM.

Albeit highly effective, peptide L7 exhibited limited solubility in aqueous buffer (<10 mg/mL). To enhance the aqueous solubility of L7 without compromising its efficacy, we compared the sequences of the corresponding regions in PD-L1 across various mammalian species to identify the conserved and variable residues. We next synthesized analogues of L7 in which a non-conserved hydrophobic or β-branching residue was substituted by a more hydrophilic one [17], focusing specifically on Phe^6^ and Thr^11^. The functional screening of the resulting L7 analogs (Figure 2A) led to the identification of several peptides, including A2, A8, B6, and B7, which exhibited enhanced solubility while maintaining the same or higher efficacy compared to the parent L7 peptide (Figure 2B). Notably, peptide B7 or L7N, with an Asn for Thr^11^ substitution, demonstrated the highest efficacy in reactivating PD-1-suppressed T cells (Figure 2B). To confirm this finding, a dose–response experiment was conducted with L7N, revealing an optimal concentration of 10 μM for this peptide (Figure 2C). The peptide solution became cloudy at 25 μM and above, suggesting that the dose-dependent effect of L7N was likely dictated by its aqueous solubility (Figure 2C). Nevertheless, L7N exhibited no significant toxicity to cells even at higher concentrations (Figure S3). Mechanistically, we found that peptide L7N, but not a control peptide with a scrambled sequence or vehicle, effectively blocked the PD-1/PD-L1 interaction in the coculture (Figure 2D). Collectively, these data suggest that peptide L7N effectively activated T cells by blocking the PD-1/PD-L1 interaction.

### 3.3. L7N and a Lipid Chimera of the Peptide Effectively Activated T Cells in Multiple Systems

**Figure 3:**
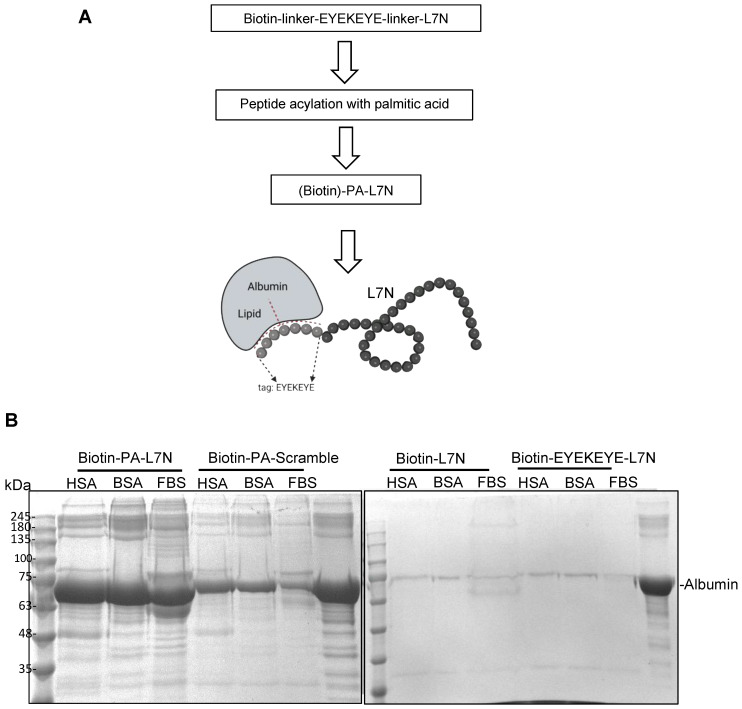
Stabilization of L7N via generation of an albumin-binding analogue. (**A**) A schematic showing the main steps taken for synthesizing a palmitic acid–peptide chimera of L7N, or PA-L7N, for albumin binding. An analogue based on scrambled L7N was included as control (PA–scramble). Two types of linkers were used. For pulldown studies, 6-aminohexanoic acid (ahx) was used as the linker, and the peptide was synthesized with a biotin at the N-terminus. Alternatively, a polyethylene glycol linker was used (refer to Table 1 for details). (**B**) PA-L7N with biotin labeling effectively pulled down albumin in vitro. (Left) Equal concentrations (0.03 mM) of human serum albumin (HSA), bovine serum albumin (BSA), or fetal bovine serum (FBS) were incubated with 1 mM of the biotin–PA-L7N peptide or the biotin–PA–scrambled peptide. Streptavidin beads were used to pull down the biotin peptides, and the bound albumin was detected by SDS-PAGE. (Right) HSA, BSA, and FBS showed no binding to biotin–L7N (with or without the EYEKEYE tag).

Although peptides may demonstrate favorable stability in vitro, issues such as rapid clearance and enzymatic degradation hinder their efficacy in vivo, primarily owing to their small molecular size. Various strategies have been reported to enhance the in vivo stability of peptides [18,19,20]. One such strategy involves shielding peptides with albumin [15], a protein renowned for its exceptional stability and low immunogenicity in vivo. We adopted the same strategy to create a version of L7N with enhanced stability to facilitate subsequent experiments in mice. Specifically, a peptide tag (EYEKEYE) was added to the N-terminus of L7N, and through the sidechain amine group of the Lys residue within the tag, palmitic acid (PA) was then coupled to the peptide, yielding the PA-L7N chimera (Figure 3A). All the synthesized peptides were purified by HPLC; the identity of the peptides was validated by mass spectrometry (Table 1). Palmitic acid (PA) has been shown to bind albumin with high affinity [15]. To confirm the binding of PA-conjugated peptides to albumin, biotinylated versions of these and control peptides were synthesized (Table 1) and then employed to capture albumin from various sources. We found that PA-L7N and, to a lesser extent, the PA-coupled scramble peptide, but not L7N or tag-L7N (without PA), bound effectively to albumin (Figure 3B).

**Figure 4:**
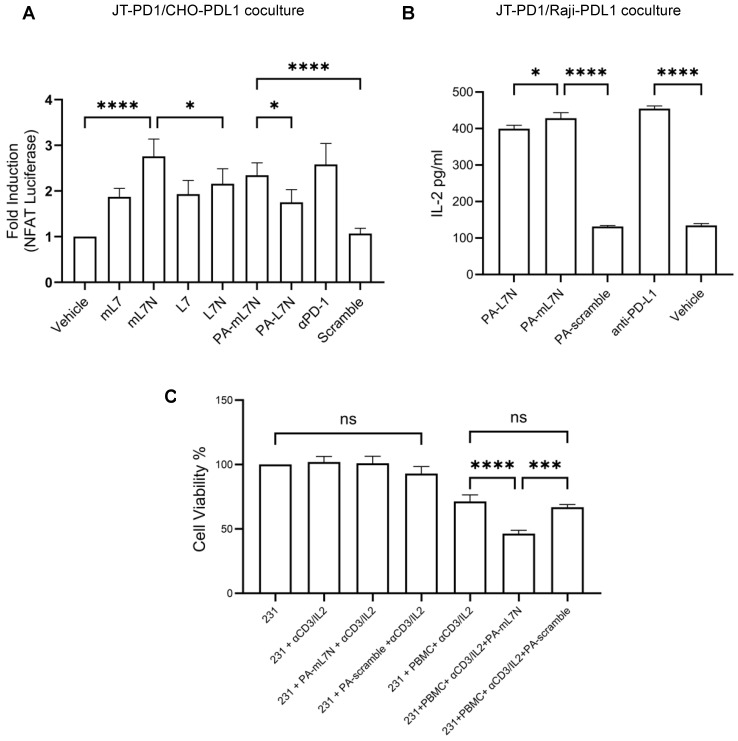
Evaluation of the efficacy of L7 and L7 analogues in reinvigorating PD-1-exhausted T cells and promoting cancer cell killing by PBMCs. (**A**) The indicated peptides (10 μM) were added to the JT-PD1/CHO-PDL1 coculture, followed by NFAT luciferase assays to determine T-cell activation. A PD-L1 antibody was included for comparison. (**B**) The PA-tagged versions of both L7N and mL7N significantly reinvigorated T cells in the JT-PD1/Raji-PDL1 coculture. Shown are IL-2 levels 24 h post addition of the peptides or antibody. (**C**) PA-mL7N significantly promoted killing of MDA-MB-231 cells by PBMCs ex vivo while exhibiting no apparent toxicity to the cancer cells at 10 μM. Shown are cell viability data of MDA-MB-231 cells under the indicated conditions measured by WST-8 assay. *, *p* < 0.01; ***, *p* < 0.0001; ****, *p* < 0.00001; one-way ANOVA test.

Previous studies [21,22,23] showed that both mouse and human PD-L1 could engage PD-1 from either species with comparable efficiency, suggesting that human and mouse PD-L1 may be functionally exchangeable. We conducted a comprehensive evaluation of the L7 and L7N series peptides, including both the human and mouse versions, the corresponding PA chimeras, or scrambled control peptides, in various coculture systems (Figure 4 and Figure S4). Compared to the scrambled mL7N peptide or vehicle, both the mouse and human L7, L7N, and PA-L7N peptides significantly enhanced the NFAT luciferase signal in the JT-PD1/CHO-PDL1 coculture. Intriguingly, mL7N/PA-mL7N appeared to be more effective than human counterparts in this assay (Figure 4A and Figure S4B). We repeated the experiment utilizing the Jurkat T cell–Raji B cell coculture, commonly used to study T cell–APC interactions [24]. To facilitate screening for PD-1/PD-L1 blockers, we cocultured JT-PD1 cells with PD-L1 overexpression Raji B cells (Raji-PDL1). The interaction of T and B cells was facilitated by the addition of the SEE bacterial superantigen which serves to engage the TCR molecules in JT-PD1 cells with the MHC molecules in Raji-PDL1 cells [25]. When the assay was performed in the presence of peptide blockers or anti-PD-L1 antibody, we found that PA-mL7N significantly outperformed PA-L7N or the PA-mL7N-scramble in enhancing IL-2 secretion by the T cells (Figure 4B). To interrogate if PA-mL7N/L7N would also increase the activity of cytotoxic T cells, we employed peripheral blood-derived mononuclear cells to kill the MDA-MB-231 breast cancer cells in the presence of anti-CD3 antibody. While PA-mL7N/L7N exhibited no effect on the viability of the cancer cells in the absence of PBMCs, the addition of either peptide led to a significant decrease in the viability of MDA-MB-231 (231 in short) cells (Figure 4C and Figure S5). Collectively, the results from these functional studies highlight the therapeutic potential of the L7N and mL7N peptides. The demonstrated efficacy of the PA-mL7N/mL7N peptides in reinvigorating T cells and enhancing the cytotoxic activity of PBMC against cancer cells suggests a potential for these peptides as novel immune-modulatory agents.

**Figure 5:**
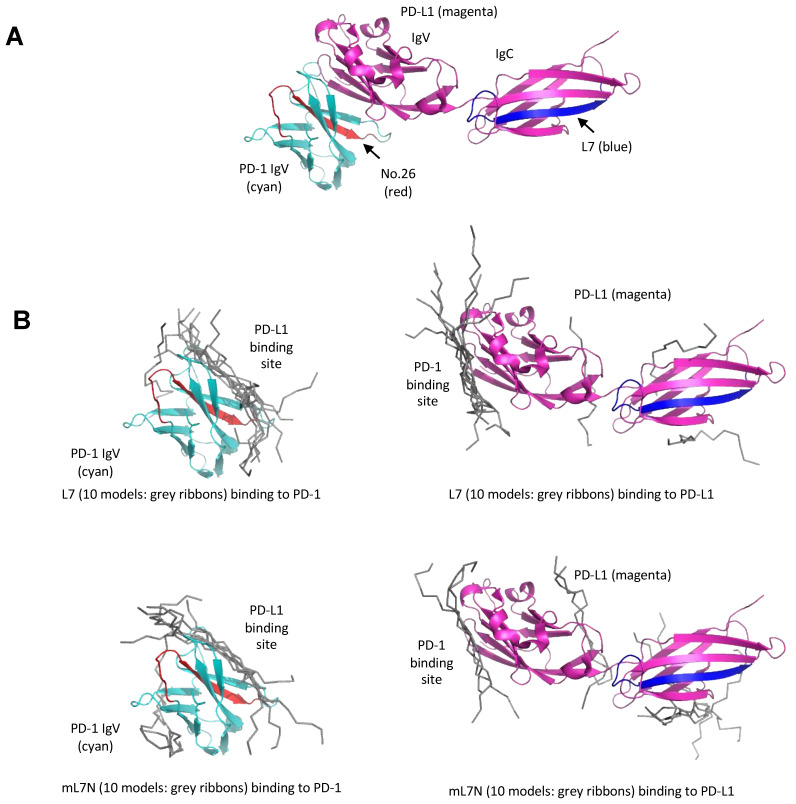
Models for binding of the peptide blockers to the PD-1/PD-L1 ectodomains. (**A**) Peptide #26 is located at the interface between the PD-1 and PD-L1 IgV domains. The structure shown is a composite of the complex structure between the PD-1 and PD-L1 IgV domains (PDB code: 4ZQK). The PD-L1 structure shown contains both the IgV and IgC domains (PDB code: 4Z18). PD-1 is in cyan, while PD-L1 is in magenta. The region corresponding to the PD-1-derived peptide #26 (PD-1 Ser57-Ser71) is shown in orange red. The region corresponding to PD-L1-derived L7 peptide (PD-L1 Arg186-Thr201) is shown in blue. (**B**) Docking of the L7 or mL7N peptide to either the PD-1 or PD-L1 ectodomain. The HPEPDOCK server was used for prediction. The top 10 predicted peptide models from each complex calculation are shown in grey ribbons. The L7 and mL7N peptides were predicted to bind similarly to the PD-1 or PD-L1 IgV domains, which resemble each other in structure. The predicted binding site for L7/mL7N is the interface between PD-1 and PD-L1, as shown in (**A**).

Computational modeling suggests that peptide #26, which is derived from the PD-1 IgV domain, is located at the binding interface between the PD-1 and PD-L1 IgV domains. Therefore, peptide #26, which was not studied in depth herein due to prior studies on a peptide inhibitor with an overlapping sequence [16], may block the PD-1/PD-L1 interaction by direct competition for binding to the PD-L1 IgV domain (Figure 5A). While peptides L7/mL7N demonstrated remarkable efficacy in in vitro models, the underlying mechanisms of their action are not as apparent as for peptide #26. Notably, the L7 peptide is a part of the IgC domain of PD-L1 that is not involved in direct binding to PD-1 [26] (Figure 5A). To gain insight into the underlying molecular mechanism, we employed the HPEPDOCK server to predict the binding modes between L7/mL7N and PD-1/PD-L1. Intriguingly, we found that both peptides were docked at the binding interface of the two proteins, suggesting that L7/mL7N could bind directly to the PD-1/PD-L1 IgV domains which are similar in structure (Figure 5B).

### 3.4. PA-mL7N Effectively Curbed Tumor Growth in Mice by Blocking the PD-1/PD-L1 Interaction and Promoting Tumor Recruitment of CD8+ T Cells

**Figure 6:**
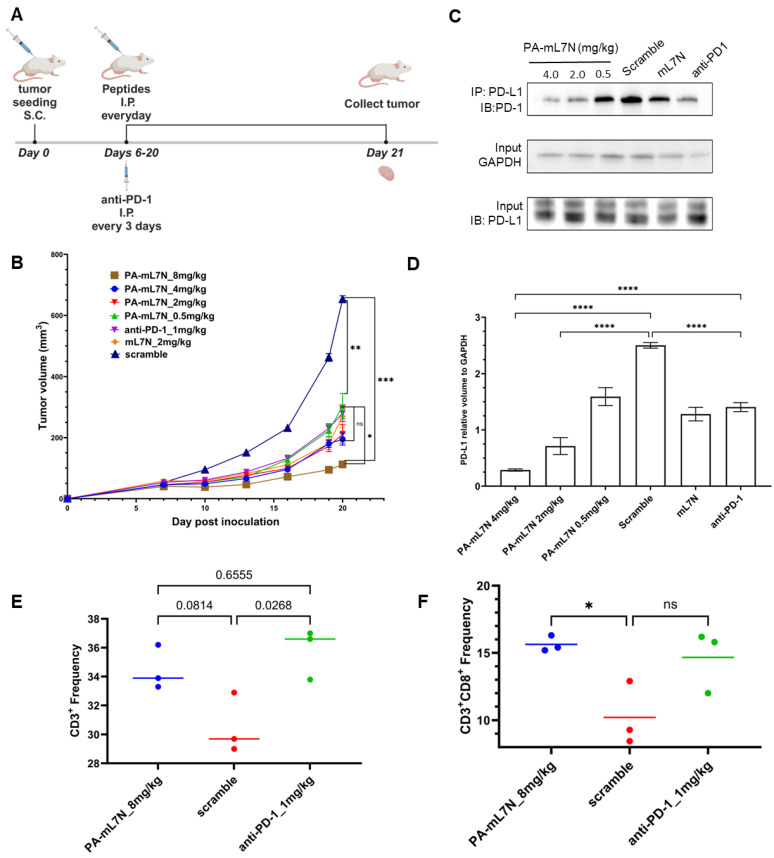
Peptide PA-mL7N effectively slowed down tumour growth in mice. (**A**) PA-mL7N, administered at 0.5–8 mgs/kg, was evaluated on 4T1-bearing BALB/c mice in comparison to an anti-PD-1 antibody (1 mg/kg, i.p). (**B**) Tumor growth curves for mice treated with the indicated peptides or αPD-1. (**C**) Western blot on tumours harvested on day 21 to show reduced binding of PD-1 to PD-L1 in tumors treated with increasing concentrations of the PA-mL7N peptide. (**D**) Quantification of the Western blot data (*n* = 3) in (**C**). (**E**,**F**) Flow cytometry data showing frequency of recruitment of total T cells (CD45+CD3+, (**E**)) or CD8+ T cells (CD45+CD3+CD8+, (**F**)) to tumours treated with PA-mL7N, the scrambled control, or αPD-1. *, *p* < 0.05; **, *p* < 0.01; ***, *p* < 0.0001; ****, *p* < 0.00001; one-way ANOVA.

The in vivo evaluation of the peptide blockers was conducted on a 4T1 syngeneic mouse breast cancer model [27]. Due to the adverse immune reactions of mice to human L7N, the mouse experiments were focused on evaluating the therapeutic potential of PA-mL7N and mL7N in comparison to anti-PD-1. Four dosages of PA-mL7N, ranging from 0.5 to 8 mg/kg, were tested along with a scrambled control peptide and mL7N, each administered at 2 mg/kg, and an PD-1 antibody (anti-PD-1) at 1 mg/kg. The peptides were administered daily, whereas the antibody was administered every three days via intraperitoneal (I.P.) injections. Tumors were collected on day 21 for Western blot (WB) and flow cytometry analyses (Figure 6A).

A dose-dependent anti-tumor effect was observed for PA-mL7N wherein, at 0.5–4 mg/kg, the peptide significantly reduced tumor progression compared to mice treated with the scrambled peptide. No significant difference was observed in anti-tumor efficacy between anti-PD-1 (at 1 mg/kg) and the PA-mL7N peptide at 0.5–4 mg/kg or mL7N at 2 mg/kg. However, at 8 mg/kg, PA-mL7N significantly outperformed the PD-1 antibody (Figure 6B). At the treatment endpoint, tumors were excised for IP/WB analysis to assess the PD-1/PD-L1 interaction. As shown in Figure 6C, PA-mL7N, but not the scrambled peptide, inhibited PD-1 binding to PD-L1 in a dose-dependent manner. At 4 mg/kg, the PA-mL7N peptide blocked the PD-1/PD-L1 interaction significantly more effectively than anti-PD-1 (Figure 6D).

The excised tumors were also processed into single-cell suspensions for analysis by flow cytometry. Antibodies specific to CD45+, CD3+, and CD8+ were employed to delineate distinct T-cell populations within the tumor microenvironment. The cells were gated for CD45+ to identify immune cells and subsequently for CD3+ to identify T cells. As shown in Figure 6, the PA-mL7N peptide resulted in an increase in tumor-infiltrating T cells (TILs, CD3+), but the corresponding p value did not reach the significant threshold (*p* < 0.05). In contrast, αPD-1 treatment led to a significant increase in TILs, yet there was no significant difference between αPD-1 and PA-mL7N (Figure 6E). These observations notwithstanding, we observed a significant increase in tumor-infiltrating CD8+ T cells in tumors treated with PA-mL7N (at 8 mg/kg) but not in tumors treated with the anti-PD-1 antibody (Figure 6F). These data suggest that PA-mL7N may exert its anti-tumor effect by promoting tumor cell killing by CD8+ cytotoxic T cells. 

## 4. Discussion

The identification of L7N/mL7N as highly effective peptides in modulating the PD-1/PD-L1 immune checkpoint in vitro and curbing tumor growth in vivo underscores the importance of combining unbiased functional screening with rational peptide design. Our screen started with assessing overlapping peptides representing the ectodomains of PD-1 or PD-1 in reactivating PD-1-suppressed T cells. The resulting hit peptides were optimized through additional rounds of peptide array screening focused on the hit peptides and flanking regions. To improve on the aqueous solubility of the peptide blockers, we replaced the less conserved, potentially non-essential hydrophobic residues with more hydrophilic amino acids. At each step, the resulting peptides were evaluated by functional assays to gauge their ability to reinvigorate exhausted T cells. Distinct from many previous studies that were focused on identifying high-affinity binding peptides for PD-1 or PD-L1 [9,13,28], our work was directed at identifying peptides capable of reactivating PD-1-suppressed T cells in several T cell–antigen presenting cell or target cell coculture systems. While peptide L7N, derived from human PD-L1, exhibited favorable characteristics in the T-cell reactivation functional assays, its high potential to generate an unintended immune response and be rapidly removed from circulation due to degradation or renal clearance [29] necessitated further improvements. We addressed these issues by replacing the human L7N with the mouse version, mL7N. Moreover, the mL7N peptide was modified to carry a palmitic acid moiety, allowing albumin binding and the protection of the peptide in vivo. These meticulous efforts culminated in the development of PA-mL7N as an effective peptide-based immune modulator. That PA-mL7N significantly outperformed anti-PD-1 in curbing tumor growth in the 4T1 syngeneic mouse breast tumor model by blocking the PD-1/PD-L1 interaction and promoting CD8+ T-cell recruitment highlights its potential as a peptide immune checkpoint blocker for cancer treatment. It is important to note that mL7N compared favorably to several published peptide blockers of PD-1/PD-L1. Specifically, OPBP-1 [30] demonstrated good efficacy in promoting T-cell proliferation at a concentration of 100 µM in in vitro cocultures. In contrast, L7N exhibited significantly higher efficacy at a lower concentration of 10 µM. Although PA-mL7N required a higher dosage in vivo and the effective dose for OPBP-1 was 0.5 mg/kg, the two peptides were tested in different tumor models, making direct comparisons between PA-mL7N and OPBP-1 difficult. Another peptide, MOPD1 [31], also showed excellent inhibitory activity against human PD-1/PD-L1 and mouse PD-1/PD-L1 interactions in vitro, with an IC50 of 4.84 µM, slightly better than L7N/mL7N. In vivo, MOPD1 was tested at dosages of 0.5 mg/kg and 2 mg/kg, but neither dosage showed better efficacy than the anti-PD-L1 antibody. In conclusion, L7N, mL7N, and PA-mL7N are effective PD-1/PD-L1-blocking peptides with therapeutic potential.

How does a small peptide block the PD-1/PD-L1 interaction? Our computational modeling suggests that both peptide #26 and L7/mL7N may bind directly to PD-1 and/or PD-L1 to block their interaction (Figure 5).

It is also likely that L7 and mL7N, especially the latter, may interfere with the PD-1-PD-L1 interaction via an indirect mechanism. The PD-L1 IgC domain contains three Asn residues—N192, N200, and N219—which can undergo N-glycosylation in cells [32,33]. N-glycosylation has been shown to protect PD-L1 from GSK3β-induced degradation [34]. Intriguingly, the L7 peptide encompasses two of these Asn residues, N192 and N200. The L7N peptide analogue contains an additional Asn substitution in place of a Thr residue in L7. It is tempting to speculate that L7/L7N/mL7N may affect the N-glycosylation and, thereby, the structure and function of PD-L1. Nevertheless, L7N/PA-mL7N effectively blocked the PD-1-PD-L1 interaction in vitro and in vivo, suggesting a significant role for the IgC domain in the PD-1/PD-L1 interaction. Supporting this notion, a recent study on CD80 and CD86 (ligands for CD28 and CTLA-4) demonstrated that the removal of their respective IgC domain markedly reduced receptor binding affinity and impaired T-cell co-stimulation signals [35]. 

To enhance the translational potential of the peptide immune checkpoint blockers, further studies are required to define the pharmacokinetics and biodistribution of the lead peptides, especially PA-mL7N, in multiple tumor models in immunocompetent mice. Additionally, exploring the long-term effects of peptide administration and potential immunogenic responses in vivo would contribute to a more thorough safety assessment. In conclusion, our study offers a multifaceted exploration of the design, optimization, and validation of peptides targeting the PD-1/PD-L1 immune checkpoint signaling pathway. The findings from our study establish a framework for the exploration of peptide-based immunomodulatory agents with potential applications in treating cancer and other conditions characterized by dysregulated immune responses. 

## Figures and Tables

**Table 1:** Sequences of peptide blockers characterized in this study.

Peptide ID	Sequence
L7	R E E K L F N V T S T L R I N T
L7 scramble	T K L R E I F V N L R T N S E T
L7N	R E E K L F N V T S N L R I N T
L7N scramble	T K L R E I F V N L R T N S E N
Biotin-L7N	Biotin-ahx-R E E K L F N V T S N L R I N T
Biotin-EYEKEYE-L7N	Biotin-ahx-E Y E K E Y E-ahx-R E E K L F N V T S N L R I N T
Biotin-PA-L7N	Biotin-ahx-E Y E K(palmitic acid) E Y E-ahx-R E E K L F N V T S N L R I N T
Biotin-PA-scramble	Biotin-ahx-E Y E K(palmitic acid) E Y E-ahx-T K L R E I F V N L R T N S E N
mL7N	T E G M L L N V T S N L R V N A
mL7N scramble	R N A L G T L E V M T N L S V N
PA-L7N	E Y E K(palmitic acid) E Y E (PEG)2 (PEG)2 (PEG)2 R E E K L F N V T S N L R I N T
PA-L7N scramble	E Y E K(palmitic acid) E Y E (PEG)2 (PEG)2 (PEG)2 T K L R E I F V N L R T N S E N
PA-mL7N	E Y E K(palmitic acid) E Y E (PEG)2 (PEG)2 (PEG)2 T E G M L L N V T S N L R V N A
PA-mL7N scramble	E Y E K(palmitic acid) E Y E (PEG)2 (PEG)2 (PEG)2 R N A L G T L E V M T N L S V N
^L^P-1	FPNWSLRPMNQM

## Data Availability

Original data available upon request.

## Supplementary Materials

The following supporting information can be downloaded at https://www.mdpi.com/article/10.3390/cells13141193/s1, Figure S1: Identification of peptide hits from the PD-1 ectodomain by peptide-walking array and T cell activation screen. Figure S2: Screening of a truncation peptide array of PD-1 hit peptide No.12 yielded the minimum sequence for activity. Figure S3:Peptide L7N was not significantly toxic to cells. Figure S4: Evaluation of the efficacy of mL7N (mouse) and human L7N in re-invigorating PD-1- suppressed T cells. Figure S5: Evaluation of PA-L7N efficacy in promoting cancer cell-killing by PBMCs.
