# Supplementary material for: Peptide Blockers of PD-1-PD-L1 Interaction Reinvigorate PD-1-Suppressed T Cells and Curb Tumor Growth in Mice
Source: Cells. 2024 Jul 15;13(14):1193. doi: 10.3390/cells13141193 (PMC11274521; doi:10.3390/cells13141193)
Supplement: Supplementary file 1 [file cells-13-01193-s001.zip › cells-3019093-supplementary.pdf]

Supplementary Figures to Zhong et al.

Peptide blockers of PD-1-PD-L1 interaction reinvigorate PD-1-suppressed T cells and curb tumor growth in mice

A

MQIPQAPWPV VWAVLQLGWR PGWFLDSPDR PWNPPTFSPA LLVVTEGDNA 50
TFTCSFSNTS ESFVLNWYRM SPSNQTDKLA AFPEDRSQPG QDCRFRVTQL 100
PNGRDFHMSV VRARRNDSGT YLCGAISLAP KAQIKESLRA ELRVTERRAE 150
VPTAHPSPSP RPAGQFQTLV VGVVGGLLGS LVLLVWVLAV I

B

1	Q-I-P-Q-A-P-W-P-V-V-W-A-V-L-Q-L-G-W	19	D-C-R-F-R-V-T-Q-L-P-N-G-R-D-F-H-M-S
2	P-W-P-V-V-W-A-V-L-Q-L-G-W-R-P-G-W-F	20	V-T-Q-L-P-N-G-R-D-F-H-M-S-V-V-R-A-R
3	W-A-V-L-Q-L-G-W-R-P-G-W-F-L-D-S-P-D	21	N-G-R-D-F-H-M-S-V-V-R-A-R-R-N-D-S-G
4	L-G-W-R-P-G-W-F-L-D-S-P-D-R-P-W-N-P	22	H-M-S-V-V-R-A-R-R-N-D-S-G-T-Y-L-C-G
5	G-W-F-L-D-S-P-D-R-P-W-N-P-P-T-F-S-P	23	R-A-R-R-N-D-S-G-T-Y-L-C-G-A-I-S-L-A
6	S-P-D-R-P-W-N-P-P-T-F-S-P-A-L-L-V-V	24	D-S-G-T-Y-L-C-G-A-I-S-L-A-P-K-A-Q-I
7	W-N-P-P-T-F-S-P-A-L-L-V-V-T-E-G-D-N	25	L-C-G-A-I-S-L-A-P-K-A-Q-I-K-E-S-L-R
8	F-S-P-A-L-L-V-V-T-E-G-D-N-A-T-F-T-C	26	S-L-A-P-K-A-Q-I-K-E-S-L-R-A-E-L-R-V
9	L-V-V-T-E-G-D-N-A-T-F-T-C-S-F-S-N-T	27	A-Q-I-K-E-S-L-R-A-E-L-R-V-T-E-R-R-A
10	G-D-N-A-T-F-T-C-S-F-S-N-T-S-E-S-F-V	28	S-L-R-A-E-L-R-V-T-E-R-R-A-E-V-P-T-A
11	F-T-C-S-F-S-N-T-S-E-S-F-V-L-N-W-Y-R	29	L-R-V-T-E-R-R-A-E-V-P-T-A-H-P-S-P-S
12	S-N-T-S-E-S-F-V-L-N-W-Y-R-M-S-P-S-N	30	R-R-A-E-V-P-T-A-H-P-S-P-S-P-R-P-A-G
13	S-F-V-L-N-W-Y-R-M-S-P-S-N-Q-T-D-K-L	31	P-T-A-H-P-S-P-S-P-R-P-A-G-Q-F-Q-T-L
14	W-Y-R-M-S-P-S-N-Q-T-D-K-L-A-A-F-P-E	32	S-P-S-P-R-P-A-G-Q-F-Q-T-L-V-V-G-V-V
15	P-S-N-Q-T-D-K-L-A-A-F-P-E-D-R-S-Q-P	33	P-A-G-Q-F-Q-T-L-V-V-G-V-V-G-G-L-L-G
16	D-K-L-A-A-F-P-E-D-R-S-Q-P-G-Q-D-C-R	34	Q-T-L-V-V-G-V-V-G-G-L-L-G-S-L-V-L-L
17	F-P-E-D-R-S-Q-P-G-Q-D-C-R-F-R-V-T-Q	35	G-V-V-G-G-L-L-G-S-L-V-L-L-V-W-V-L-A
18	S-Q-P-G-Q-D-C-R-F-R-V-T-Q-L-P-N-G-R	36	V-G-G-L-L-G-S-L-V-L-L-V-W-V-L-A-V-I

C

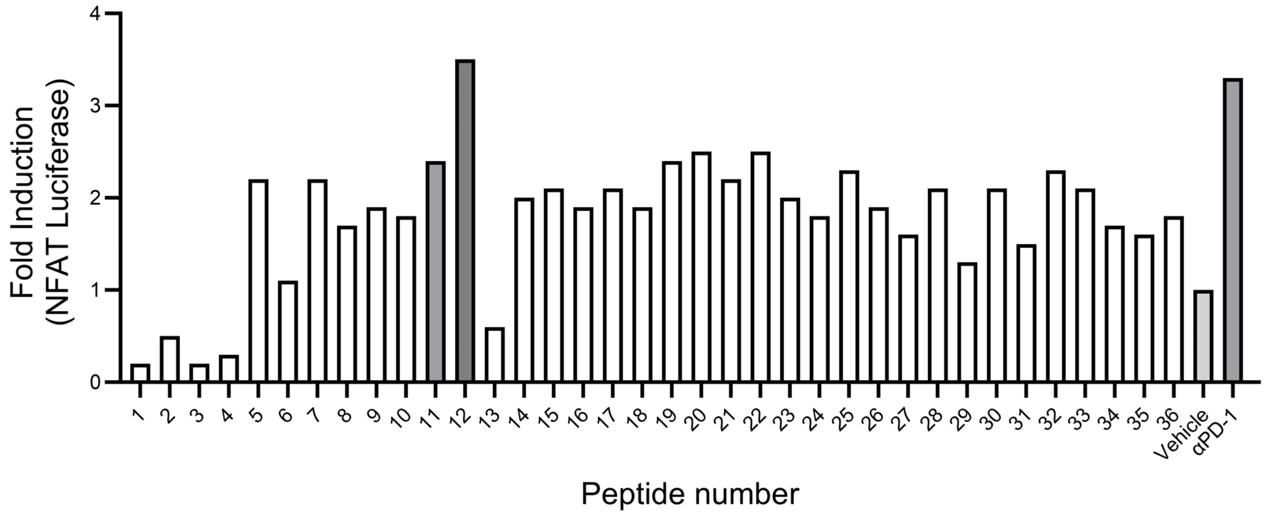

Figure S1. Identification of peptide hits from the PD-1 ectodomain by peptide-walking array and T cell activation screen. (**A**) Amino acid sequence of the human PD-1 extracellular and transmembrane regions. Residues involved in binding PD-L1 were highlighted in yellow. The underlined sequence corresponded to peptide No.12 in the walking array below. (**B**) Sequences of peptides in the PD-1 walking array (18mer peptides with 5 aa overlap between consecutive peptides) encompassing the region shown in (A). (**C**) Functional screening of the synthesized peptides (200μM) in (B) using the Jurkat-PD-1/CHO-PD-L1 coculture. Shown are fold changes in NTAF-luciferase signals produced by individual peptides relative to the vehicle (DMSO) . An anti-PD-1 antibody (αPD-1, 2.5 μg/ml) was included as a positive control.

A

1	L-V-V-T-E-G-D-N-A-T-F-T-C-S-F-S-N-T	24	S-N-T-S-E-S-F-V-L-N-W-Y-R-M-S-P-S
2	V-T-E-G-D-N-A-T-F-T-C-S-F-S-N-T-S-E	25	S-N-T-S-E-S-F-V-L-N-W-Y-R-M-S-P
3	E-G-D-N-A-T-F-T-C-S-F-S-N-T-S-E-S-F	26	S-N-T-S-E-S-F-V-L-N-W-Y-R-M-S
4	G-D-N-A-T-F-T-C-S-F-S-N-T-S-E-S-F-V	27	S-N-T-S-E-S-F-V-L-N-W-Y-R-M
5	N-A-T-F-T-C-S-F-S-N-T-S-E-S-F-V-L-N	28	S-N-T-S-E-S-F-V-L-N-W-Y-R
6	F-T-C-S-F-S-N-T-S-E-S-F-V-L-N-W-Y-R	29	S-N-T-S-E-S-F-V-L-N-W-Y
7	C-S-F-S-N-T-S-E-S-F-V-L-N-W-Y-R-M-S	30	S-N-T-S-E-S-F-V-L-N-W
8	F-S-N-T-S-E-S-F-V-L-N-W-Y-R-M-S-P-S	31	S-N-T-S-E-S-F-V-L-N
9	S-N-T-S-E-S-F-V-L-N-W-Y-R-M-S-P-S-N	32	S-N-T-S-E-S-F-V-L
10	S-F-V-L-N-W-Y-R-M-S-P-S-N-Q-T-D-K-L	33	S-N-T-S-E-S-F-V
11	N-T-S-E-S-F-V-L-N-W-Y-R-M-S-P-S-N	34	S-N-T-S-E-S-F
12	T-S-E-S-F-V-L-N-W-Y-R-M-S-P-S-N	35	S-N-T-S-E-S
13	S-E-S-F-V-L-N-W-Y-R-M-S-P-S-N	36	S-N-T-S-E
14	E-S-F-V-L-N-W-Y-R-M-S-P-S-N	37	A-N-T-S-E-S-F-V-L-N-W
15	S-F-V-L-N-W-Y-R-M-S-P-S-N	38	S-A-T-S-E-S-F-V-L-N-W
16	F-V-L-N-W-Y-R-M-S-P-S-N	39	S-N-A-S-E-S-F-V-L-N-W
17	V-L-N-W-Y-R-M-S-P-S-N	40	S-N-T-A-E-S-F-V-L-N-W
18	L-N-W-Y-R-M-S-P-S-N	41	S-N-T-S-A-S-F-V-L-N-W
19	N-W-Y-R-M-S-P-S-N	42	S-N-T-S-E-A-F-V-L-N-W
20	W-Y-R-M-S-P-S-N	43	S-N-T-S-E-S-A-V-L-N-W
21	Y-R-M-S-P-S-N		
22	R-M-S-P-S-N		
23	M-S-P-S-N		

B

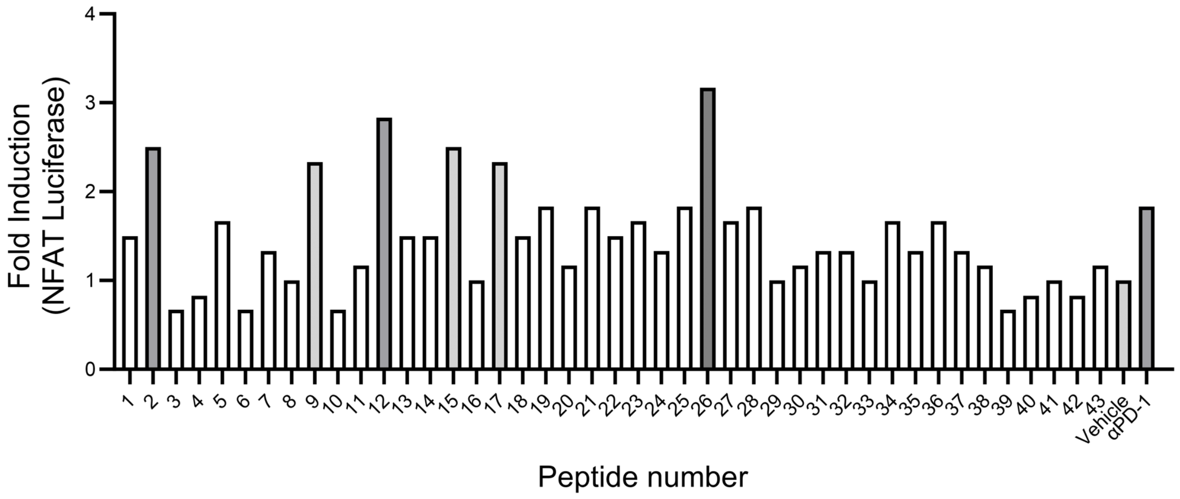

Figure S2. Screening of a truncation peptide array of PD-1 hit peptide No.12 yielded the minimum sequence for activity. (**A**) A secondary array of peptides targeting the hit peptide No. 12 (highlighted in yellow) identified from the primary screen in Fig. S1. Peptides #1-#10 represented a finer peptide-walking array series (16mer with 3 aa overlap between consecutive peptides) of the region surrounding peptide hit No. 12. Peptides #11-23 represented the N-terminal truncation series while peptides #24-#36 the C-terminal truncation series of peptide No. 12. Peptides #37-#43 were Ala-scanning analogues of peptide #30 (identified in green). (**B**) Functional screening of the synthesized peptides (200μM) in (A) using the Jurkat-PD-1/CHO-PD-L1 coculture. Shown are fold changes in NTAF-luciferase signals produced by individual peptides relative to the vehicle (DMSO). An anti-PD-1 antibody (αPD-1, 2.5 μg/ml) was included as positive control.

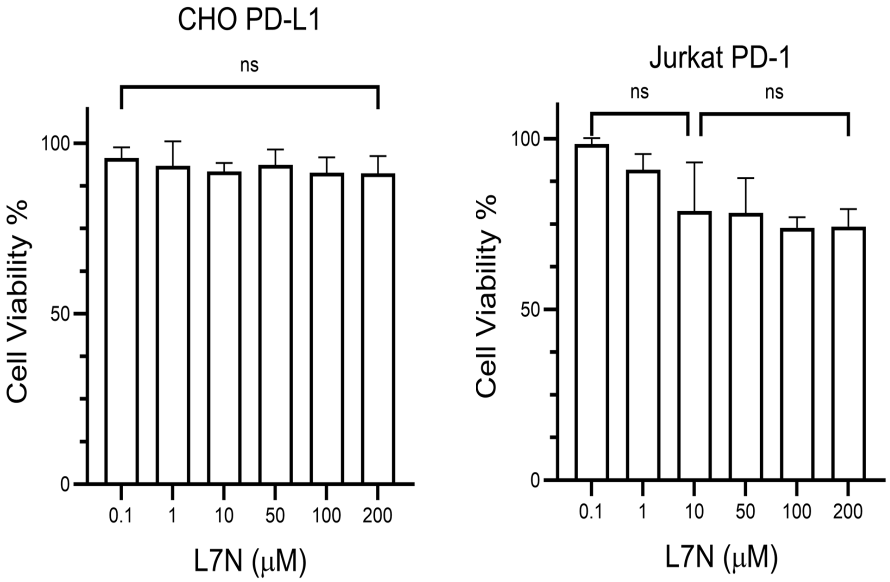

Figure S3. Peptide L7N was not significantly toxic to cells. Shown are percentages of viable cells (relative to vehicle treated cells, set at 100%), calculated based on WST-8 assays carried out on CHO-PDL1 or Jurkat-PD1 cells in the presence of increasing concentrations of L7N. ns, not significant, One-way ANOVA test**.**

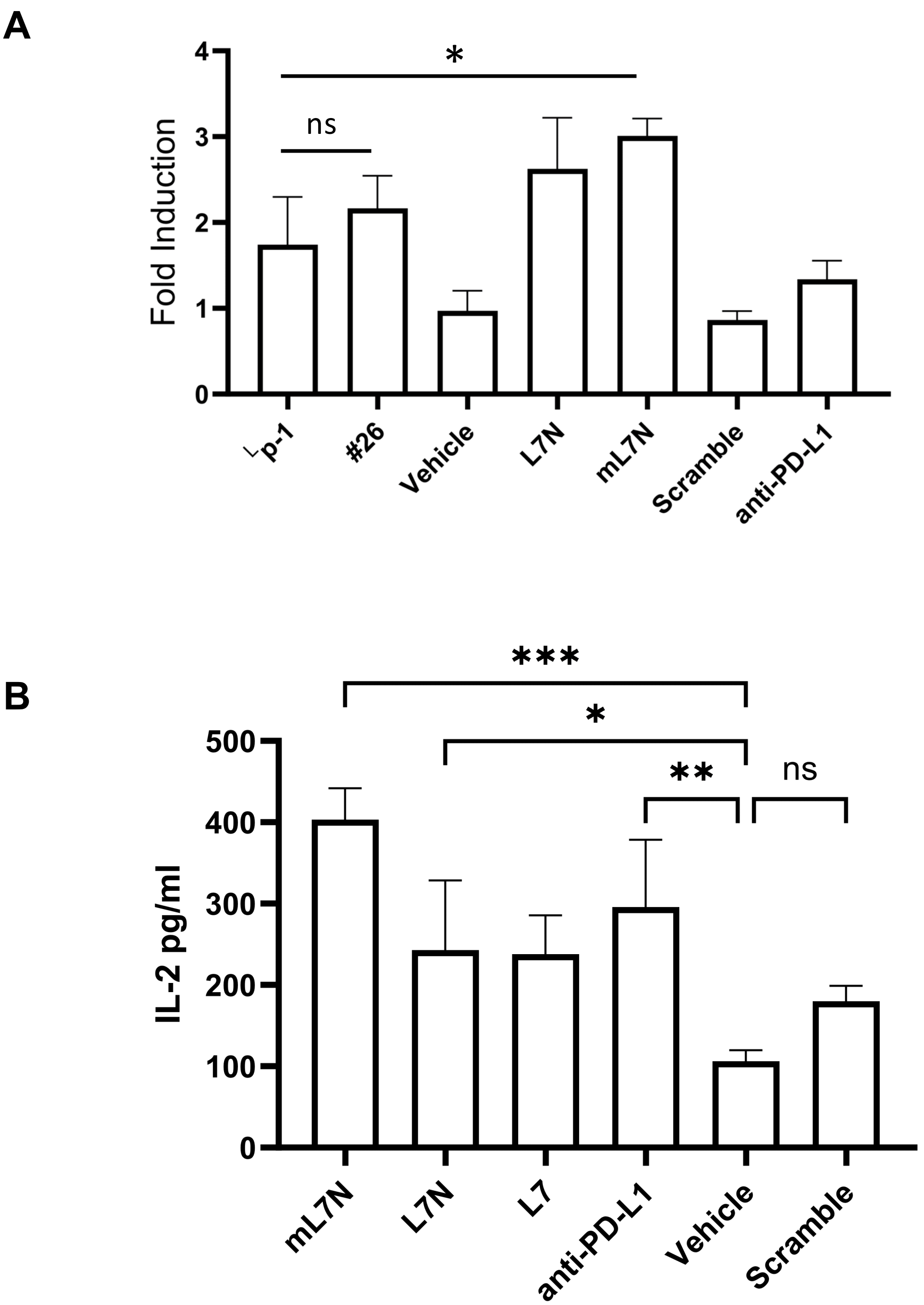

Figure S4. Evaluation of the efficacy of mL7N (mouse) and human L7N in re-invigorating PD-1-suppressed T cells. (**A**) Changes in NTAF-luciferase signals produced by individual peptides (10μM) in the JT-PD1/MDA-MB-231 coculture. A PD-L1 antibody (anti-PD-L1, 5 μg/ml) and a scrambled mL7N control peptide were included for comparison. The peptide ^{L}P-1 was based on Chang et al (2015, reference #10). Peptide #26 correspond to the top hit identified in Fig.S2 from the PD-1 ectodomain screen. (**B**) Peptides L7N and mL7N reinvigorated T cells in the JT-PD1/Raji-PDL1 coculture. Shown are IL-2 levels in 24 hours post addition of the peptides or antibody. *, $p<0.01$. **, $p<0.001$. ***, $p<0.0001$, One-way ANOVA test.

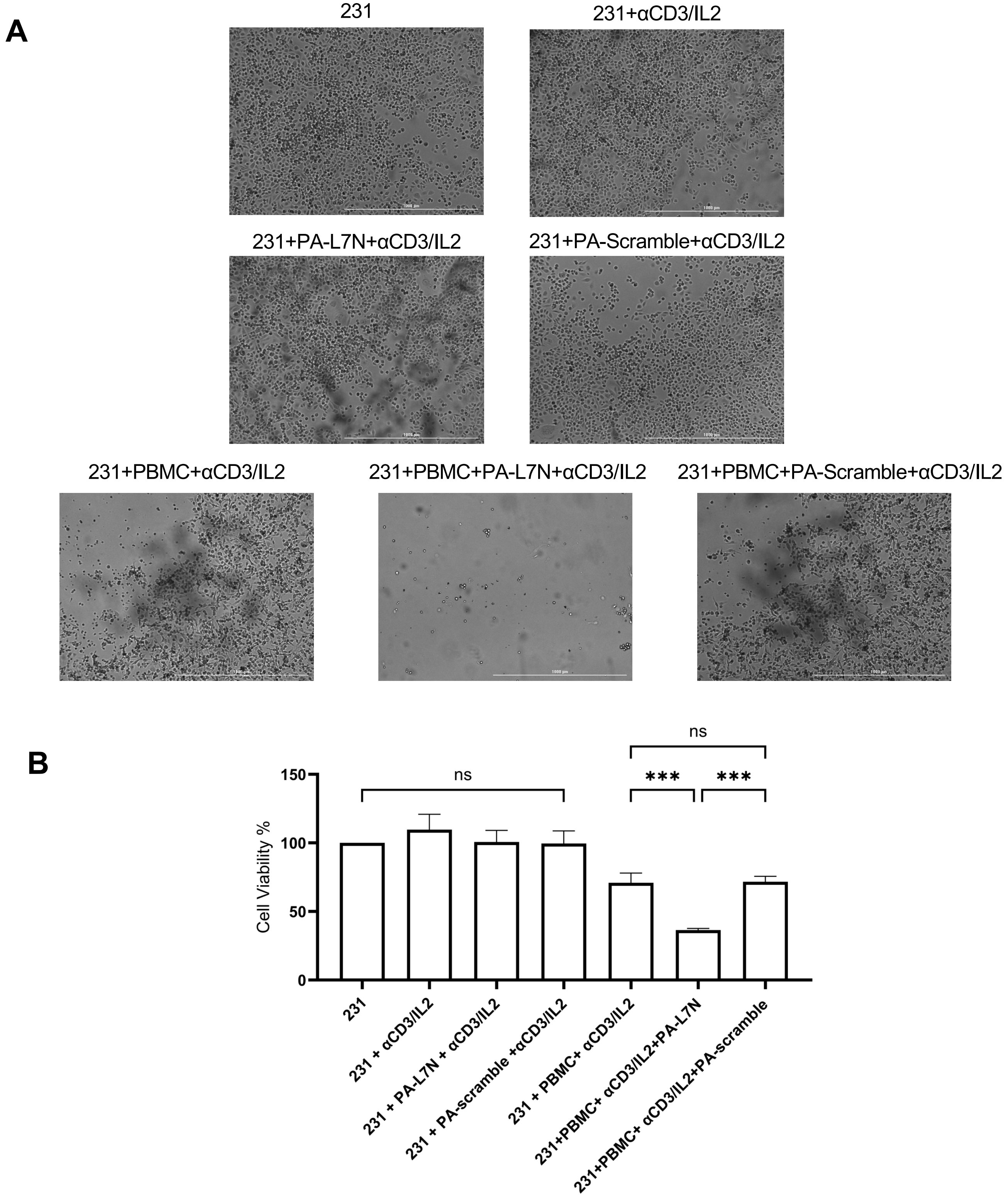

Figure S5. Evaluation of PA-L7N efficacy in promoting cancer cell-killing by PBMCs. (**A**) Bright-filed microscopic images of MDA-MB-231 (231) cultured with or without peripheral blood mononuclear cells (PBMCs) in the presence of the indicated peptides. PA-L7N, but not the scrambled control peptide (PA-Scarmble) significantly promoted PBMC killing of MDA-MB-231 cells ex vivo while exhibiting no apparent toxicity to the cancer cells at 10μM. (**B**) Viability data of MDA-MB-231 cells under the indicated conditions measured by WST-8 assay. *, p<0.01. **, p<0.001. ***, p<0.0001, ****, p< 0.00001, One-way ANOVA test.
